# Knowledge, attitude, and practice of medical students regarding smoking and substance abuse, Cairo University, Egypt

**DOI:** 10.1186/s42506-019-0011-z

**Published:** 2019-02-26

**Authors:** Silvia Farouk Shalaby, Mona Adel Soliman

**Affiliations:** 1 Public Health and Community Medicine Department, Faculty of Medicine, Cairo University, 131 Eltiar Fekri Street, Giza, Egypt; 2 Public Health and Community Medicine Department, Faculty of Medicine, Cairo University, 8th District, Madinet Nasr, Cairo, Egypt

**Keywords:** Smoking, Substance abuse, Faculty students

## Abstract

**Background:**

Involving medical personnel in all aspects of smoking control in the community is indispensable. In a trial to enhance the participation of healthcare professionals in smoking cessation activities, this study was conducted to evaluate knowledge, behavior, and attitude of medical school students regarding smoking and substance abuse. Perception of their future role “as physicians” in combating smoking and substance abuse was also explored.

**Subjects and methods:**

A cross-sectional descriptive study was conducted**.** A self-administered questionnaire based on standardized questionnaires prepared by the World Health Organization covering sections about knowledge, beliefs, and practices of the students regarding smoking and substance abuse was submitted to 296 students enrolled in the Faculty of Medicine of Cairo University, during the academic year 2014–2015.

**Results:**

Most of the participants had correct knowledge about health hazards of smoking, where 83.4–93.6% correctly selected the answers, but still stated that they are in need for courses about this issue. Positive attitudes were also expressed towards smoking legislations and tobacco control policies. Cigarette and shisha smoking, bango, and addictive medications abuse were low among the studied group (13.5, 15.2, 2, 3–6.4%, respectively).

**Conclusion and recommendations:**

The prevalence of smoking and substance abuse was relatively low among Cairo University medical students who had generally correct knowledge about the hazards of these practices and positive attitude towards their future role in helping their patients to quit. It may be appropriate to train students about stress management skills through organizing regular “stress coping strategies” sessions to assist them to cope with various stressors and consider implementing counseling programs to support students, especially medical students and the future doctors, who have a leading role in combating smoking and substance abuse in the community.

## Introduction

Tobacco smoking is one of the leading avoidable causes of premature death, illness, and disability all over the world [1]. Tobacco use has shown to be the sixth of eight leading causes of death worldwide [2]. An estimated 4.9 million deaths occurring every year can be linked to tobacco use. This is subjective to be increased by 10 million by the year 2020, if the current tobacco use epidemic goes on and even more than two-thirds of these deaths are expected to happen in developing countries [3]. A study conducted in Delta Region, Egypt, where 1715 medical students were questioned about knowledge and awareness about smoking and substance abuse, showed that 5.6 and 1.2% of the students reported being smokers and ex-smokers, respectively, with a higher prevalence among 6th year students, and 40% of them reported to be involved with substance abuse [4].

Medical students who are the coming physicians play an essential role in smoking prevention and control measures. Unfortunately, a lot of evidence reveals that prevalence of smoking tobacco is fairly elevated among medical students. As shown by a study, one out of every three medical students in their last year of medical school, who currently smoke, started after entering medical school. The smoking habits of medical students are subject to the same phenomena that affect those of the general population of this age group [5].

Experts have suggested that undergraduates at medical schools have to be armored with information and skills to enhance smoking quitting behaviors among their patients in the future [6–8]. A study conducted in Australia reported that although progress has been made to address the teaching of tobacco in medical schools worldwide, there is a great deal more effort required so that education on tobacco becomes an ongoing part of medical curricula. [9].

Physicians are generally viewed as individuals from whom advice on smoking would be most accepted by both smokers and non-smokers. There is an urgent requirement to reduce this harmful habit through more comprehensive public health initiatives, provision of support for cessation among health professionals who smoke, and providing them with training to allow them to be able to help their patients with cessation. [10].

Thus, among the strategies to reduce smoking-related morbidity and mortality is to promote the involvement of health professionals in tobacco-use prevention and quitting counseling [11]. Medical personnel who smoke are more likely to reveal attitudes that render them away from providing their patients with antismoking advice [12].

Drug abuse has social, physical, psychological, and economic serious impacts that in addition to personal damage, it imposes heavy costs on individuals, families, and society [13], implying a great need for health professionals to identify and treat substance abusing or addiction. The role of hospitals and other medical institutions is crucial in promoting healthy behavior in the community. Health professionals themselves play a particularly important role in tobacco control [14].

This study aimed to explore knowledge, attitude, and behavior of medical students in Cairo University about smoking and substance abuse.

## Participants and methods

A cross-sectional study was conducted among 269 students enrolled in the Faculty of Medicine of Cairo University during the academic year 2014–2015. The sample size was calculated to be 273 according to the statistical website “Raosoft” based on the following parameters: 5% margin of error, 95% level of confidence, and 62% of the students’ perspectives towards physicians’ training to be able to help smokers to quit [15]. An additional 20% was added to compensate for the possible non-response; hence, the sample size is 328. They were selected using a cluster sampling technique from the hospital rounds. Non-response rate is 9.8%.

A self-administered questionnaire in English was developed after studying the current literature and was based on guidelines and standardized questionnaires prepared by the World Health Organization (WHO) [16]. The questionnaire contained questions covering knowledge and attitude about smoking and substance abuse and also data about current and future smoking status and drug abuse. Knowledge and attitudes about the responsibilities of health sector workers in this matter were also inquired.

The questionnaire included as well information about students’ smoking and drug abuse practices (in the study, the prevalence of cigarette smoking is a lifetime use prevalence of cigarette smoking (i.e., those who ever smoked)).

Nicotine dependence was calculated according to the Fagerstrom Test for Nicotine Dependence. The test comprises of six items including how frequent the person smokes in the morning and how earlier he smokes after waking up, number of cigarettes per day, difficulty to refrain from smoking in places where forbidden or despite being ill, and the cigarette that the smoker hate most to give up.

The higher the number of cigarettes per day or in the morning, the earlier the first cigarette after waking up and the more the attachment of the smoker to cigarettes in the morning or in spite of opposing conditions; the higher is the plasma levels of nicotine and cotinine—the major metabolite of nicotine, the more is the withdrawal symptoms and the patient’s nicotine dependence.

The score ranges from 0 to 10, and scoring was done according to the following scale [17]: 1–2 means very low dependence, 3 means low dependence, 4 means moderate dependence, and 5+ means high dependence.

### Statistical analysis

Data was coded, entered, and analyzed using the Statistical Package for Social Sciences (SPSS), version18 (released in 2009, PASW Statistics for Windows, Version 18.0, SPSS Inc., Chicago). Descriptive analyses were done to summarize information by calculating the number and percent for categorical variables, whereas the mean and standard deviation (SD) was calculated for continuous variables.

## Results

Nearly equal percentage of male and female participants shared in the study (44.6% males versus 56.4% females), with mean age of 23.5 years.

Table 1 shows knowledge of students regarding health hazards of smoking and addictive effect of different substances. The majority of the participants (80.1–98%) correctly identified the health hazards of smoking. Regarding the harms of hook smoking (shisha smoking) versus harms of cigarette smoking, the majority of the participants (83.1%) stated that it is not less harmful.

**Table 1:** Knowledge of students regarding health hazards of tobacco smoking and addictive effect of different substances, Cairo University, academic year 2014–2015

	Correct		Incorrect		I do not know	
	*N*	%	*N*	%	*N*	%
Tobacco smoking health hazards						
Bladder cancer	247	83.4	17	5.7	32	10.8
Coronary artery disease	290	98.0	2	0.7	4	1.4
Lung cancer	290	98.0	2	0.7	4	1.4
Chronic bronchitis	281	95.0	9	3.0	6	2.0
Sexual dysfunction	265	89.5	10	3.4	21	7.1
Oral cancer	281	95.0	8	2.6	7	2.4
Emphysema	270	91.2	14	4.7	12	4.1
Laryngeal cancer	284	95.9	4	1.4	8	2.7
Peripheral vascular disease	273	92.2	10	3.4	13	4.4
Leukoplakia	237	80.1	15	5.0	44	14.9
Neonatal mortality	261	88.1	12	4.1	23	7.8
Cerebrovascular disease	277	93.6	6	2.0	13	4.4
Shisha smoking harm in comparison to cigarette smoking						
Is shisha smoking less harmful than cigarette smoking?	246	83.1	39	13.2	11	3.7
Addiction causation by addictive substances						
Tobacco smoking	205	69.2	78	26.4	13	4.4
Bango	252	85.1	34	11.5	10	3.4
Codeine	272	91.9	11	3.7	13	4.4
Xanax	175	59.1	21	7.1	100	33.8
Valium	206	69.6	41	13.8	49	16.6
Tramadol (Tramal)	272	91.9	12	4.1	12	4.1

As for addictive substances, about 85–92% stated that bango, codeine, and tramadol have an addictive effect, while only 59.1–69.6% of the participants identified tobacco, Xanax, and valium as addictive substances.

Regarding the participant's attitude towards the effect of smoking, as shown in Table 2, 82.5% of the study participants who have ever smoked believe that smoking is harmful to health. Also about 70 to 87% of the study participants disagreed and strongly disagreed about any assumed benefits for smoking. Regarding the causes of non-smoking or quitting smoking, the main causes were health protection and self-discipline followed by other causes such as causing no harm to their families, setting an example for their patients and society, or saving money.

**Table 2:** Attitudes of students regarding the effect of smoking, causes of non-smoking, and legislations for banning it, Cairo University, academic year 2014–2015

Attitude	Strongly agree		Agree		Disagree		Strongly disagree	
	*N*	%	*N*	%	*N*	%	*N*	%
Smoking is harmful	30	75	3	7.5	6	15	1	2.5
Assumed benefits of smoking								
Relief stress	25	8.4	65	22.0	109	36.8	97	32.8
Give self-confidence	11	3.7	46	15.5	108	36.5	131	44.3
Increase concentration	13	4.4	51	17.2	111	37.5	121	40.9
Social benefits	7	2.4	32	10.8	92	31.1	165	55.7
Causes of non-smoking								
Health protection	206	82.1	39	15.5	2	0.8	4	1.6
Self-discipline	177	70.5	61	24.3	6	2.4	7	2.8
Symptoms related to smoking	157	62.5	67	26.7	18	7.2	9	3.6
Avoid discomfort to others	144	57.4	74	29.5	24	9.6	9	3.6
To save money	128	51.0	69	27.5	40	15.9	14	5.6
Example for children	147	58.6	71	28.3	20	8.0	13	5.2
Example for patients	148	59.0	67	26.7	22	8.8	14	5.6
Example socially	148	59.0	69	27.5	22	8.8	12	4.8
Not to harm the family	157	62.5	58	23.1	23	9.2	13	5.2
Pressure from colleagues	89	35.5	46	18.3	78	31.1	38	15.1
Example to health workers	129	51.4	62	24.7	40	15.9	20	8.0
Pressure from the family	94	37.5	47	18.7	74	29.5	36	14.3
Legislations for banning smoking								
Smoking in closed public places should be prohibited or restricted to certain area.	212	71.6	68	23.0	9	3.0	7	2.4
The selling price of tobacco products should increase sharply.	162	54.7	74	25.0	40	13.5	20	6.8
Smoking in hospitals should be restricted to special smoking areas.	182	61.4	65	22.0	26	8.8	23	7.8
Smoking in hospitals should be totally banned.	213	72.0	54	18.2	21	7.1	8	2.7
Smoking-free hospitals provides better quality health services.	191	64.5	59	19.9	24	8.1	22	7.4
There should be programs to help healthcare workers to quit.	187	63.2	96	32.4	6	2.0	7	2.4
The faculty should adopt programs that help medical students to quit smoking.	189	63.8	87	29.4	15	5.1	5	1.7
Smoking of doctors could affect the behavior of others.	173	58.4	99	33.4	18	6.2	6	2.0
Smoking in movies and series should be restricted.	151	51.0	93	31.4	39	13.2	13	4.4

As for smoking banning legislations, about 80–96% of the participants agreed and strongly agreed about lobbying for different legislations, restrictions, and quitting programs to limit smoking including banning of smoking advertisements. Also, they agreed that smoking-free hospitals provide better quality of healthcare.

The attitudes of students regarding the role of physicians in combating smoking and the sufficiency of their knowledge regarding antismoking activities are shown in Table 3. Positive attitudes of students about their exemplary role in combating smoking were high; upon asking the participants about the activities they believe they should share in as a part of their vocational responsibility, about 92.2% stated that they ought to persuade their patients in every possible opportunity to quit smoking.

**Table 3:** Attitudes of students regarding the role of physicians in combating smoking and the sufficiency of their knowledge regarding antismoking activities, Cairo University, academic year 2014–2015

Attitude	Strongly agree		Agree		Disagree		Strongly disagree	
	*N*	%	*N*	%	*N*	%	*N*	%
It is the responsibility of doctors to convince people to stop smoking.	104	35.1	148	50.0	37	12.5	7	2.4
Most smokers can stop smoking if they want to.	117	39.5	130	43.9	38	12.8	11	3.7
Doctors should set a good example by not smoking.	181	61.1	97	32.8	12	4.1	6	2.0
Most smokers are not willing to stop smoking despite the physician’s advice.	101	34.1	152	51.4	29	9.8	14	4.7
Doctors should go beyond the activity of diffusing knowledge about the hazards of smoking, if you agree.	120	40.5	142	48.0	29	9.8	5	1.7
On every appropriate occasion, you should persuade your patient to quit smoking.	88	29.7	185	62.5	19	6.4	4	1.4
Your present knowledge is sufficient to persuade patients to quit smoking.	62	20.9	162	54.7	69	23.3	3	1.0
Health professionals should get specific training on how to help patients who want to stop smoking.	149	50.3	129	43.6	13	4.4	5	1.7

Despite that, about three-quarters of the respondents believed that they have sufficient information to persuade patients to stop smoking, yet more than 90% of them agreed that physicians should receive special training courses to be able to help smokers to quit smoking.

Table 4 shows the patterns and frequency of smoking among participants: The percent of study participants who have ever smoked cigarette was 13.5%. Out of the 40 smokers, 30 were males and 10 were females. The mean age for starting smoking was 18.1 years old (SD ± 3.1). About two-thirds of the smokers stated that they have started smoking after being enrolled at the Faculty of Medicine. When asked about their willingness to quit smoking, 67.5% stated that they want to stop smoking while 75% of them said they had previous serious attempts to stop smoking.

**Table 4:** Patterns and frequency of smoking and bango use among students, Cairo University, academic year 2014–2015

Patterns	Frequency	Percentage
Cigarette smoking		
Have you ever smoked cigarettes		
Yes	40^#^	13.5
No	256	86.5
At what age have you started smoking?		
Range, Mean ± SD	10–24	18.1 ± 3.1
Median, IQR	18.0 (16.3–20.0)	
Did you smoke within the previous 30 days?		
Yes	21	52.5
No	19	47.5
How many cigarettes do you smoke/day		
Range, Mean ± SD	2–60	16.6 ± 14.8
Median, IQR	12.5 (6.5–20.0)	
How many months have passed since you last smoked?		
Range, Mean ± SD	1–19	6.4 ± 6.2
Median, IQR	4.5 (1.5–10.5)	
Did you start to smoke after you joined your medical school?		
Yes	26	65.0
No	14	35.0
Do you want to stop smoking?		
Yes	27	67.5
No	13	32.5
Have you ever made a serious attempt to stop smoking?		
Yes	30	75.0
No	10	25.0
May you smoke in presence of your patients?		
Yes	24	60.0
No	11	27.5
I do not know	5	12.5
How soon after you wake up do you smoke your first cigarette?		
Within 5 min	5	12.5
Within 6–30 min	9	22.5
Within 31–60 min	10	25.0
After 60 min	16	40.0
Do you find it difficult to avoid smoke cigarettes in places where it is forbidden (e.g., at the library, in cinema)?		
Yes	13	32.5
No	27	67.5
Which cigarette would you hate most to give up?		
The first one in the morning	14	35.0
Any other	26	65.0
How many cigarettes per day do you smoke?		
10 or less	17	42.5
11–20	9	22.5
21–30	12	30.0
31 or more	2	5.0
Do you smoke more during the first hours after waking than during the rest of the day?		
Yes	14	35.0
No	26	65.0
Do you smoke even when you are ill enough to be in bed most of the day?		
Yes	16	40.0
No	24	60.0
Shisha smoking		
Have you ever smoked shisha?		
Yes	45	15.2
No	251	84.8
Did you smoke shisha in the previous 30 days?		
Yes	24	53.3
No	21	46.7
How many times do you or did you smoke shisha per week?		
Range, Mean ± SD	1–10	3.8 ± 3.1
Median, IQR	3.0 (1.0–6.3)	
How many months have passed since you last smoked?		
Range, Mean ± SD	1–12	4.8 ± 3.5
Median, IQR	4.0 (2.5–7.0)	
Bango		
Have you ever used Bango?		
Yes	6	2.0
No	290	98.0
Did you use bango in the previous 30 days?		
Yes	4	66.7
No	2	33.3

^#^30 males and 10 females

With regard to smoking in front of their patients, 60% stated that they would do that while 12.5% did not know what would be their behavior at that time.

Regarding hook (shisha) smoking, 15.2% of the participants have tried shisha before and the mean of shisha smoking times per week was 3.8 ± 3.1. In addition, 2% of the participants stated using cannabis, and out of them, 66.7% stated using cannabis in 30 days preceding the study.

Table 5 shows the levels of nicotine dependence among the smokers, and low level of dependence was found among 67.5% of smokers while high level of dependence was found among 22.5% of smokers.

**Table 5:** Levels of nicotine dependence among the smoking students, Cairo University, academic year 2014–2015

Levels of nicotine dependence	Frequency (*n* = 40)	Percentage
Nicotine dependence		
Very low (0–2)	14	35.0
Low (3–4)	13	32.5
Medium (5)	4	10.0
High (6–7)	7	17.5
Very high (8–10)	2	5.0
Nicotine dependence		
Range, Mean ± SD	0–9	3.5 ± 2.3
Median, IQR	3.0 (2.0–5.0)	

NB: The nicotine dependence index was calculated according to the Fagerstrom Test for Nicotine Dependence, 2010 [17]. The number between brackets is the interquartile range

Table 6 shows the frequency of addictive medication abuse among participants. 9.1% of the participants have used one or more addictive medications without medical prescription. Medications such as Xanax, tramadol, and valium were used by 4.4–6.4% of the research subjects. Out of them, 4.1% have used one of the abovementioned drugs at least once in 30 days preceding the study and the frequency of intake was 4.9 ± 5.1 per week.

**Table 6:** Addictive medications abuse among students, Cairo University, academic year 2014–2015

Addictive medications abuse	Yes		No	
	*N*	%	*N*	%
Have you ever used any addictive medications without medical prescription?	27	9.1	269	90.9
Have you ever used any of the following medications without medical prescription?				
Xanax	19	6.4	277	93.6
Valium	14	4.7	282	95.3
Tramadol (Tramal)	13	4.4	283	95.6
Other addictive medications	9	3.0	287	97.0
Did you use any of the above drugs within the previous 30 days?	12	4.1	284	95.9
How many times do you or did you use per week?				
Range, Mean ± SD	1–15		4.9 ± 5.1	
Median, IQR	3.0 (1.0–8.8)			

The displayed results of addictive medications use are not mutually exclusive

## Discussion

The studied sample had generally correct information about smoking health hazards. Similar results were shown by medical students of Agha-Khan University, Karachi, Pakistan, who expressed correct knowledge when asked about dangers of smoking, and they have also suggested that physicians and medical students should have proper smoking cessation training courses [18].

The mean age of starting smoking in the current study was 18.1 ± 3.1 years. In Iran, a study about smoking behaviors of medical students [19] showed that the mean age of smoking was 19.6 ± 2.5 and 18.9 ± 2.4 years for male and female students, respectively. Initiating smoking at this age may be due to causes related to relieving distress and pleasure or due to social causes as displayed by the current study and Iran study.

Regarding the harms of hook smoking versus harms of cigarette smoking, the majority of the participants stated that it is not less harmless. A study conducted in Malaysia among young people showed that 57.3% of the participants agreed that shisha use exposes the smoker to large amounts of smoke while the majority was uncertain about the hazards of shisha smoking compared to tobacco smoking [15].

The prevalence of smoking among medical students in Cairo University (13%) was nearly similar to the prevalence of smoking among students in Saudi Arabian medical schools where the prevalence was 14.3%. [17] The prevalence was lower than that among the medical students of other Arabic countries such as Bahrain (27.5%), Jordan (26.3%), Yemen (27.0%), and Syria (15.8%) [20–22].

Smoking among females was found to be much less when compared to males, a result that goes in accordance with a study in Pakistan, which showed lower smoking prevalence among females. This finding may be due to similar cultural factors as smoking is considered as a taboo, so females especially young women rarely smoke or never exposed their smoking habit to society [23].

It is generally accepted that physicians are like role models to patients, so their attitude and behavior about smoking represent a major influence on the psychology of the patients regarding this issue. Among medical students, health-related behaviors and hygiene practices have a greater influence on their academic performances and future professional prospects [24].

In the current study, the participants had positive attitude towards banning smoking by law, results that goes in accordance with a study done in Riyadh, KSA, where students showed positive attitude towards minimizing passive smoking through their support of banning smoking in public areas as well as their willingness to discuss and advise their patients to quit smoking. [25]. This attitude may stem from the satisfactory level of awareness of the participants regarding health hazards of smoking, similar to a study conducted among medical students in Turkey which showed that among the smokers, 92.6% considered smoking harmful to health, 81.5% worried that smoking was harmful to their health, but 12.3% did not worry about it [26].

Medical students have distinct stressors and predispositions for drug abuse. Pressure to achieve good grades is often thought to be a powerful driver for drug use [27]. In the present study, drug abuse behavior constituted a small percentage among the participants, a finding that was different from a study conducted in a medical college in India that explored drug abuse behavior among graduate medical students that constituted 20.43% of 230 participants [27].

A recent review on substance abuse among medical students in the USA noted that there were so few studies in this area. [28]. Available research, however, indicates that the actual rate of drug use in medical schools in the USA has been lower than that of an equivalent non-medical school population and this abuse started before students began their medical studies. The authors considered that the stress of medical school may not be a major factor as was originally hypothesized. [28] The rate of abuse in the present study is lower than those reported in the USA (10%) [29] and India (20%) [30]. The latter high rate may be related to the fact that it included various substances used by the participants including alcohol, cigarettes, cannabis, bhang, tobacco (chewing), and other substances (gel and drugs). A lower rate of drug use (3.9%) was reported in a study among male students in different faculties in Isfahan and Kermanshah medical universities in Iran. The rate was relatively lower than other substance abuse (especially smoking (19.4%) and alcohol drinking (10.1%,) [31].

## Conclusion and recommendations

The prevalence of smoking and drug abuse was relatively low among Cairo University medical students who had generally correct knowledge about the hazards of these practices. Their perceptions about their future role as doctors towards smoking control were promising. They showed positive supportive attitudes towards tobacco banning legislations and were enthusiastic to receive more training that would help them in their tasks as physicians. Medical students ought to be educated about avoiding such behaviors under any circumstances and to be trained about stress management skills without having to smoke or abuse drugs. In this aspect, different and regular “stress coping strategies” sessions could be organized for medical students to assist them to cope with various stressors.

Drug abuse of substances such as cannabis and other medications ranged from 2–6.4% among the studied sample. It may be appropriate to consider implementing counseling programs to support students, especially medical students and the future doctors, who have a leading role in combating smoking and substance abuse in the community. More research is to be conducted to find the optimum way for implementation of curricula change for early prevention of smoking and to address smoking cessation programs for medical students during their study years.

### Limitations of the study

The study handled a sensitive issue that despite the questionnaire was anonymous, underreporting of tobacco smoking and substance abuse by the students could not be excluded. Another limitation is that the study was done only among Cairo University medical students, so its results cannot be generalized to other medical schools in Egypt.
